# Barriers to timely discharge of preterm infants from neonatal care: A single‐centre audit

**DOI:** 10.1111/apa.17443

**Published:** 2024-10-03

**Authors:** Janine Abramson, Tilly Bedford, Shalini Ojha, Lisa Szatkowski

**Affiliations:** ^1^ Centre for Perinatal Research School of Medicine, University of Nottingham Nottingham UK; ^2^ Neonatal Unit University Hospitals of Derby and Burton NHS Trust Derby UK

**Abbreviations**
DOL: day of life
GA: gestational age
IQR: interquartile range
NG: nasogastric
PMA: post‐menstrual age
UK: United Kingdon

Each year, approximately 1 in every 13 infants born in the UK, nearly 58 000 infants in total, are born prematurely (before 37 completed weeks of pregnancy).^1^ Many of these, including all born before 34 weeks gestational age (GA), are admitted to neonatal units for specialist care. Most go home between 37 and 40 weeks post‐menstrual age (PMA, the time elapsed between the first day of the mother's last menstrual period and the infant's current age), depending upon their GA at birth and medical needs.^2^

There are no UK guidelines for timing of discharge from neonatal care. However, ‘discharge readiness’ typically requires a level of physiological maturity such that an infant weighs at least 1700 g, can breathe without support, take all their nutritional requirements orally and maintain their own body temperature. The milestone that takes the longest to achieve is referred to as the ‘final discharge barrier’.^3^ Identifying the most common final discharge barrier, and how soon after reaching this milestone infants are discharged home, could help inform the design of strategies to facilitate safe, earlier discharge, improving infant and family well‐being and reducing health service costs.^3^

We conducted an audit in the neonatal unit at the Royal Derby Hospital, UK, including all infants born at <34 weeks' GA from 01 January 2015 to 31 December 2022 who survived to discharge home. We excluded infants who were discharged home weighing <1700 g (*n* = 14), with a nasogastric (NG) feeding tube (*n* = 128) and/or on home oxygen (*n* = 95). We used data from electronic patient records to identify the day of life and PMA when infants reached each physiological milestone and the final discharge barrier. We calculated the median (IQR) number of days delay between reaching physiological readiness and final discharge and estimated the potential cost saving if this delay could be reduced. For a 6‐week period in autumn 2022 we reviewed all paper‐based patient records, medical and nursing notes to identify factors associated with delayed discharge. The study was registered as a service evaluation and approved by the clinical audit team at University Hospitals of Derby and Burton NHS Foundation trust.

We identified 762 eligible infants, with a median (IQR) GA of 32 (30–33) weeks and birthweight of 1634 (1311–1962) grams. Achieving independent feeding was the final discharge barrier for 624 (81.9%) infants, reached on average on day 25 (17–41) of life (Table 1). Breathing independently and reaching 1700 g in weight were the final barriers for 101 (13.3%) and 69 (9.1%) infants respectively (some infants reached 2 or 3 milestones on the same day). Data on ability to maintain temperature were not routinely recorded in electronic patient records, but in our review of all paper‐based documentation, no infant had their discharge delayed due to need for additional temperature support. Overall, infants were physiologically ready for discharge on day 25 (17–43) and were discharged on day 29 (19–47), at a PMA of 35^+5^ (34^+6^–36^+5^) and 36^+1^ (35^+1^–37^+1^) weeks respectively. On average, infants were discharged 2^1^, ^2^, ^3^, ^4^ days after meeting all physiological criteria, with little variation in the delay to discharge by GA group.

**TABLE 1:** Day of life and postmenstrual age when infants reached physiological milestones, readiness for discharge and final discharge home, and estimated cost savings of reducing length of neonatal unit stay.

	All infants	22–27 weeks GA	28–31 weeks GA	32–33 weeks GA
Number of infants	762	57	318	387
DOL and PMA weight ≥ 1700 g, median (IQR)	14 (1–28) 33^+4^ (33^+0^–34^+4^)	54 (42–66) 33^+5^ (32^+6^–34^+6^)	26 (18–34) 33^+6^ (32^+5^–34^+6^)	1 (1–9) 33^+3^ (33^+0^–34^+0^)
DOL and PMA no respiratory support required, median (IQR)	13 (2–33) 33^+6^ (33^+1^–35^+1^)	77 (58–94) 37^+1^ (35^+4^–39^+1^)	27 (17–39) 34^+1^ (33^+1^–35^+4^)	3 (1–8) 33^+4^ (33^+1^–34^+1^)
DOL and PMA no feeding support required, median (IQR)	25 (17–41) 35^+4^ (34^+5^–36^+3^)	79 (65–93) 37^+2^ (36^+1^–38^+6^)	37 (28–49) 35^+4^ (34^+4^–36^+6^)	17 (13–22) 35^+3^ (34^+6^–36^+0^)
DOL and PMA physiologically ready for discharge, median (IQR)	25 (17–43) 35^+5^ (34^+6^–36^+5^)	80 (66–100) 37^+4^ (36^+4^–39^+4^)	38 (29–51) 35^+6^ (34^+5^–37^+1^)	17 (13–22) 35^+3^ (34^+6^–36^+1^)
DOL and PMA of discharge, median (IQR)	29 (19–47) 36^+1^ (35^+1^–37^+1^)	84 (67–101) 38^+0^ (36^+5^–39^+6^)	42 (31–54) 36^+2^ (35^+0^–37^+4^)	19 (15–25) 35^+6^ (35^+1^–36^+4^)
Number of additional days per infant, median (IQR, range)	2 (1–4, 0–32)	2 (0–4, 0–20)	2 (1–4, 0–32)	2 (1–3, 0–25)
Total number of additional days across all infants	2283	176	1080	1027
Total estimated cost of additional days, per year^a^	£ 207 676	£16 010	£98 244	£93 422
Total estimated cost saving by reducing stay by 1 day^b^, per year	£59 310	£3366	£26 107	£29 837

Abbreviations: DOL, day of life; PMA, postmenstrual age; IQR, interquartile range.

^a^ Based on published costs for delivering neonatal care.^5^

^b^ Among infants with 1 or more days delay.

Common diagnoses among infants experiencing a delay to discharge included suspected sepsis, jaundice, anaemia and gastro‐oesophageal reflux. Our review of paper‐based records identified social care involvement as a common reason for extended hospital stays, affecting 25% of infants over a 6‐week period, for reasons including poor maternal mental health and drug abuse, previous removal of children from mothers' care and housing concerns. Parental non‐engagement was also evident, including lack of regular visits to their child on the neonatal unit and lack of engagement with education on infant care post‐discharge. Whilst some delay to discharge after reaching physiological readiness may be entirely appropriate, our data suggest that reducing hospital stay by just 1 day could result in substantial cost savings of £59 310 per year in this one unit.

Relatively few infants are discharged home from this unit with an NG tube in place as there is only a very small home‐care nursing team to support this service. However, in many units, both in the UK and elsewhere,^4^ this is more common, as long as the infant is able to take at least some of their feeds (typically at least one quarter to one third) by breast or bottle. We did not have detailed data on feed volumes to allow us to determine when infants could safely be discharged with an NG tube in place. However, on average, infants ceased to require more invasive feeding methods (total parenteral nutrition or glucose and electrolyte infusion) on day 6 (4–11) of life and were finally discharged 9 (4–14) days after reaching this milestone alongside having met the weight and respiratory requirements for discharge. Considerable cost savings could be realised if more infants could be discharged, sooner, with an NG tube in place.

In conclusion, understanding when preterm infants are likely to be physiologically ready for discharge home from neonatal care, and reasons why their stay may be extended, is crucial for optimising resource use and alleviating infant and parental stress by reuniting families at home as soon as is safely possible. Our study highlights the need to understand both clinical and social factors to facilitate timely, safe discharge. We plan to extend this single‐centre audit to a national study to understand patterns of discharge timing and whether there are variations by neonatal unit. In this larger study we will also investigate discharge timing in infants who go home with a NG feeding tube and/or on home oxygen, to understand whether these infants can be discharged sooner once they have reached all other milestones and once the facilities for home tube feeding and breathing support are in place. The results of our national study will help us identify aspects of infant and parental care around which to design, implement and evaluate targeted interventions to support timely, safe discharge, improving outcomes for preterm infants and their families and ensuring optimal use of health service resources.

## AUTHOR CONTRIBUTIONS

**Janine Abramson:** Investigation; writing – original draft; writing – review and editing; supervision. **Tilly Bedford:** Investigation; writing – review and editing; formal analysis; data curation. **Shalini Ojha:** Conceptualization; writing – review and editing; supervision; methodology. **Lisa Szatkowski:** Writing – original draft; writing – review and editing; methodology; formal analysis; supervision; data curation; investigation.

## CONFLICT OF INTEREST STATEMENT

None to declare.

## FUNDING INFORMATION

This study did not receive any specific funding.
